# Effect of asthma control on general health-related quality of life in patients diagnosed with adult-onset asthma

**DOI:** 10.1038/s41598-019-52361-9

**Published:** 2019-11-06

**Authors:** Pinja Ilmarinen, Hind Juboori, Leena E. Tuomisto, Onni Niemelä, Harri Sintonen, Hannu Kankaanranta

**Affiliations:** 1 Department of Respiratory Medicine, Seinäjoki Central Hospital, Seinäjoki, Finland; 2 Department of Laboratory Medicine, Seinäjoki Central Hospital, Seinäjoki, Finland; 3 Faculty of Medicine and Health Technology, Tampere University, Tampere, Finland; 4 Department of Public Health, University of Helsinki, Helsinki, Finland

**Keywords:** Asthma, Respiratory signs and symptoms

## Abstract

Health-related quality of life (HRQoL) is a well-established aspect of health that can be measured by both disease-specific and general instruments. The effect of uncontrolled asthma on generic HRQoL has not been shown in patients with clinically confirmed adult-onset asthma and with asthma control defined according to the Global Initiative for Asthma, so the aim of this study was to determine this. In the 12-year follow-up cohort of the Seinäjoki Adult Asthma Study (n = 203), patients with uncontrolled and partially controlled asthma had lower generic HRQoL as determined by 15D compared to the controlled group. On 10 out of 15 dimensions of 15D, the mean scores were significantly lower in patients with uncontrolled asthma compared with those with controlled asthma. The affected dimensions were mobility, breathing, sleeping, usual activities, mental function, discomfort and symptoms, depression, distress, vitality and sexual activity. In the Tobit regression analysis, a poorer 15D score was associated with uncontrolled asthma, lower postbronchodilator FEV_1_, female sex, depression, treated dyspepsia and poorer 15D score at diagnosis. Our results show that uncontrolled asthma affects everyday life in several aspects, including previously unknown components such as sexual activity and vitality.

## Introduction

During the last decade, asthma has been revealed as a heterogeneous disease manifesting in many distinct phenotypes. A key differentiating factor is age at disease onset, separating the disease into two main categories: childhood- and adult-onset disease^1–3^. Several adult-onset phenotypes have been suggested by recent cluster analyses, e.g., late-onset eosinophilic, exercise-induced, obesity-related, neutrophilic, smoking-related and paucigranulocytic asthma^1,4^. In adult-onset disease, the therapeutic response may remain insufficient despite the use of different add-on therapies. Previous studies have shown that adult-onset asthma rarely remits^5–7^.

Quality of life is defined according to WHO as individuals’ perception of their position in life in the context of culture and value systems in which they live and in relation to their goals, expectations, standards and concerns^8^. Health-related quality of life (HRQoL) refers to how health impacts an individual’s ability to function and his or her perceived well-being in physical, mental and social domains of life^9^. HRQoL is a well-established aspect of health and general well-being that can be measured with a variety of instruments. Disease-specific HRQoL measures are designed to reflect well the HRQoL aspects of a particular disease^9,10^. Asthma-specific HRQoL refers to the perceived impact that asthma has on the patient’s quality of life^10^. Examples of HRQoL instruments used in assessing asthma-specific HRQoL are St George’s Respiratory Questionnaire (SGRQ), the Asthma Quality of Life Questionnaire (AQLQ) and its modifications and the Airways Questionnaire 20 (AQ20)^10^. However, asthma-specific HRQoL does not cover all aspects of HRQoL. In contrast, generic HRQoL instruments are designed to be applicable across a wide range of populations, diseases and interventions^9^. Examples of such instruments include the Medical Outcomes Study 36-Item Short Form (SF-36) health survey, the Nottingham Health Profile (NHP), the Sickness Impact Profile (SIP), the Health Utilities Index (HUI), the EuroQol Instrument (EQ-5D) and 15D^9,11^. The dimensions that generic HRQoL tools cover vary widely according to what aspects the tool pays attention to.

Our knowledge about how different levels of asthma control affect individual HRQoL in adults, especially using generic tools, is restricted. Few studies have concentrated on the effects of asthma control on asthma-specific HRQoL, but in these studies, the definition of asthma control has been variable and often not based on asthma control assessment as recommended by the Global Initiative for Asthma (GINA)^12–16^. Similarly, epidemiological surveys assessing asthma control with variable methods^13^, the Asthma Control Test (ACT)^14,17^ or GINA^18,19^ have proposed that uncontrolled/not well-controlled asthma may be associated with lower generic HRQoL as assessed by the SF-36^13,14,19^, KIDSCREEN^17^ or EQ-5D^18^. One previous study showed that increased severity of asthma symptoms is associated with lower generic HRQoL as measured by the 15D^20^. Overall, the findings suggest that partially controlled or uncontrolled asthma may be associated with lower generic HRQoL. Thus, the aim of the present study was to evaluate the effect of different levels of asthma control on generic HRQoL in patients with clinically confirmed adult-onset asthma and to evaluate factors related to poorer generic HRQoL.

## Methods

### Study design

**Figure 1:**
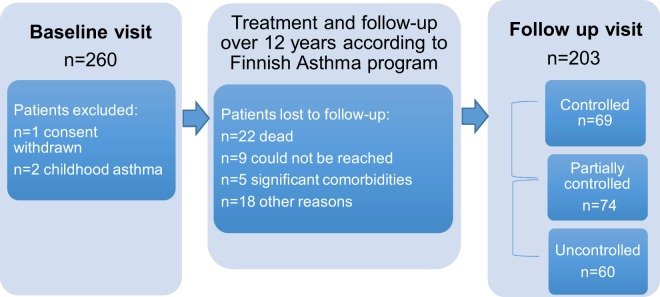
Flow chart of Seinäjoki Adult Asthma Study.

This study is a part of the Seinäjoki adult asthma study (SAAS). SAAS is a prospective, single center (Seinäjoki Central Hospital, Seinäjoki, Finland) 12-year follow-up study of a cohort of 257 patients with a new-onset asthma diagnosed at adult age (≥15 years). The study protocol and the inclusion and exclusion criteria have been previously published^21^. Asthma was diagnosed by a respiratory specialist and confirmed by objective lung function measurements (see patient selection and diagnostic criteria in Supplementary Table S1). Patients fulfilling the criteria of asthma-chronic obstructive pulmonary disease overlap (ACO) were not excluded for the cohort to be representative of true patients in the clinic. The study comprised two parts: baseline (diagnosis) and follow-up visits (Fig. 1). After the initial visit, the patients were treated for their asthma according to the Finnish Asthma Program guidelines^22^. The patients were invited to the follow-up visit 12 years after diagnosis, with a participation rate of 79% (203 patients). In this study, mainly the data collected from the follow-up visit were used. Participants gave written informed consent to the study protocol, which was approved by the Ethics Committee of Tampere University Hospital, Tampere, Finland. All methods were performed in accordance with relevant guidelines and regulations.

### Lung function and inflammatory parameters

Spirometry (Vmax Encore 22, Viasys Healthcare, Palm Springs, CA), single-breath diffusion capacity for the lungs measured using carbon monoxide (Dl_CO_) and pulmonary diffusing capacity for carbon monoxide per unit of alveolar volume (Dl_CO_/VA) were performed according to international recommendations as previously reported^4,7,23,24^. Finnish reference values for spirometry were used. The fraction of exhaled nitric oxide (FENO) was measured with a portable rapid-response chemiluminescent analyzer according to ATS standards (flow rate 50 mL/s; NIOX System, Aerocrine, Sweden). For inflammatory parameters, venous blood was collected, and white blood cells, including eosinophils, were counted. Total IgE levels were measured by using ImmunoCAP (Thermo Scientific, Uppsala, Sweden). Laboratory assays were performed in an accredited laboratory (SFS-EN ISO/IEC 17025:2005 and ISO 15189:2007) of Seinäjoki Central Hospital. Atopy was defined as at least one positive response (≥3 mm) in the skin prick test towards common aeroallergens.

### Structured questionnaires

Patients completed structured questionnaires concerning symptoms, quality of life, medication usage, background data, AQ20, ACT and 15D. The AQ20 is a short and simple questionnaire to measure and quantify disturbances in the disease-specific HRQoL of patients with asthma or chronic obstructive pulmonary disease (COPD)^25^. ACT is a widely used patient self-administered tool to assess symptoms of asthma^26^. The 15D instrument is a generic, multidimensional, standardized, self-administered evaluative tool for assessing HRQoL across 15 dimensions: mobility, vision, hearing, breathing, sleeping, eating, speech, excretion, usual activities, mental function, discomfort and symptoms, depression, distress, vitality and sexual activity. Each dimension comprises one question with five answer levels. The single index score (15D score), representing the overall HRQoL on a 0–1 scale (1 = full health, 0 = being dead) and the dimension- level values, reflecting the subject’s well-being in that dimension relative to no problems (=1) and to being dead (=0), are calculated from the health state descriptive system (questionnaire) by using a set of population-based preference or utility weights. Mean dimension-level values are used to draw 15D profiles for groups. The 15D combines the advantages of a large profile and single index score measure^11^. The minimum clinically important change or difference in the 15D score has been estimated to be ± 0.015 on the basis that people can on average feel such a difference^27^. Since it covers a wide range of dimensions and including the breathing dimension, 15D is thought to be suitable in assessing the general HRQoL in asthmatics in this study.

### Definition of asthma control

In the main analyses, the definition of asthma control was based on the GINA 2010 report^28^. Briefly, patients with uncontrolled asthma had at least 3 of the following features within 4 weeks before the 12-year follow-up visit: a) daytime symptoms more than twice a week, b) any limitation of activities, c) any nocturnal symptoms/awakenings d) need for rescue treatment more than twice a week and e) decreased lung function (FEV_1_ or PEF <80% predicted). Partially controlled patients had 1–2 of features a-e and controlled patients none. Additionally, in some analyses we used the following definitions based on GINA 2019^29^ or ACT score^26,29^:

#### GINA 2019 (symptom control)

Otherwise the same as GINA 2010 but did not include lung function (e).

#### GINA 2019 + exacerbations

Patients with uncontrolled asthma had 3–4 of features a-d. In addition, patients with <3 of the features (a–d) were considered uncontrolled if they had ≥2 exacerbations in the previous two years. Partially controlled patients had 1–2 of features a-d within 4 weeks and 0–1 exacerbations within the previous two years and controlled patients had none of features a-d within 4 weeks and 0–1 exacerbations in the previous two years.

#### ACT score

A total score of 5–15 indicated uncontrolled asthma, 16–19 partially controlled asthma, and 20–25 controlled asthma^29^.

### Statistical analysis

Continuous data are expressed as mean ± SD or median and interquartile range. When the variable was continuous, comparisons between three groups were performed by one-way ANOVA with Tukey’s post hoc test (if normally or close to normally distributed) or the Kruskal-Wallis test (if nonnormally distributed). When the variable was categorical, the χ^2^ test was used. The correlations between 15D score and variables of interest were determined by the Spearman correlation coefficient. Tobit regression (a censored regression analysis) was performed to explain the variance in the 15D score. The correlation matrix was analyzed, and the explanatory variables that were not strongly correlated (R < 0.7) were included in the analysis. Statistical analyses were performed using SPSS software, version 22 (IBM SPSS, Armonk, NY) and Stata Statistical Software, Release 15 (StataCorp 2017, Texas, TX). A *p-*value < 0.05 was regarded as statistically significant.

## Results

### Patient characteristics at follow-up

A total of 203 patients with adult-onset asthma were included. Most of them were females (58%), half were current or ex-smokers (52.7%), and a majority (76.4%) used daily inhaled corticosteroids (ICS) (Table 1)^7^. The results on lung function and inflammatory parameters have been previously published^7^.

**Table 1:** Patient characteristics at follow-up visit^a^.

	Total cohort (n = 203)
Age (y)	58 (14)
Male gender n (%)	85 (41.9)
BMI (kg/m^2^)	28.1 (24.4–31.2)
Smokers (incl.ex) n (%)*	107 (52.7)
Post-bd FEV_1_/FVC < 0.7 and pack-y ≥ 10 n (%)	34 (16.7)
Smoking history, pack-y	16 (7–30)
Total IgE (kU/l)	61 (24–163)
Daily ICS in use n (%)	155 (76.4)
Blood eosinophils (x10^9^/l)	0.17 (0.10–0.27)
Pre-bd FEV_1_%	86 (76–96)
Pre-bd FVC %	96 (87–106)
Pre-bd FEV_1_/FVC	0.73 (0.66–0.79)
Post-bd FEV_1_%	90 (80–98)
Post-bd FVC %	99 (88–107)
Post-bd FEV_1_/FVC	0.75 (0.69–0.80)
Dl_CO_, %	93 (18)
Dl_CO_/VA, %	95 (16)
AQ20 score	4 (2–7)

Shown are mean (SD) or median (25–75 percentiles). BMI = Body Mass Index, bd = Bronchodilator, FEV_1_ = Forced expiratory volume for 1 second, FVC = Forced vital capacity, Dl_CO_ = Diffusing capacity, Dl_CO_/VA = Diffusing capacity adjusted by the alveolar volume, AQ20 = Airways Questionnaire. ^a^Part of the results have been previously published^7^.

### Clinical characteristics and asthma-specific HRQoL of controlled, partly controlled and uncontrolled asthma

The basic characteristics of patients separated by the asthma control level have been previously published^7^. Briefly, patients in the groups of uncontrolled and partially controlled asthma were older, were more often smokers, had worse lung function, and used higher doses of ICS and more medications to treat comorbidities than the controlled group^7^. Both groups had lower asthma-specific HRQoL as compared to the controlled group as shown by the AQ20. In addition, patients with uncontrolled asthma had worse lung function and lower HRQoL compared to patients with partially controlled asthma, as measured by the AQ20 (Table 2)^7^.

**Table 2:** Characteristics of patients with different asthma control at follow up according to GINA 2010^a^.

	Controlled n = 69 (34%)	Partially controlled n = 74 (36%)	Uncontrolled n = 60 (30%)	p-value
Male gender n (%)	21 (30.4)	32 (43.2)	32 (53.3)*	0.030
Age at follow-up (y)	54 (14)	60 (12)*	61 (13)**	0.005
BMI (kg/m^2^)	27.7 (4.8)	28.9 (5.7)	29.1 (6.1)	0.324
Smokers (incl.ex) n (%)	25 (36.2)	45 (60.8)**	37 (61.7)**	0.003
Pack-years	7 (3–16)	18 (7–25)	29 (15–35)**^,#^	0.001
Post FEV_1_/FVC < 0.7 and pack-y ≥ 10 n (%)	5 (7.2)	10 (13.7)	19 (32.2)***	0.001
Daily ICS users n (%)	47 (68.1)	57 (77.0)	51 (85.0)	0.078
ICS dose of daily users (budesonide eq µg)	550 (400–1000)	800 (713–1000)*	1000 (400–1350)**	0.016
Daily ICS + LABA n (%)	19 (27.5)	36 (48.6)	41 (68.3)	<0.001
No of add-on drugs^&^	0 (0–1)	1 (0–1)**	1 (1–1)***^,#^	<0.001
Daily SABA use n (%)	2 (2.9)	7 (9.5)	14 (23.3)***	<0.001
No of drugs in use to treat co-morbidities	1 (0–3.5)	1 (0–3)	2 (1–6)**^,##^	0.001
Psychiatric disease n (%)	10 (14.5)	9 (12.2)	8 (13.3)	0.919
Depression n (%)	5 (7.2)	6 (8.1)	6 (10.0)	0.849
Persistent or allergic rhinitis or rhinoconjunctivitis n (%)	45 (65.2)	49 (66.2)	48 (80.0)	0.128
Total IgE (kU/l)	47 (23–134)	76 (25–164)	72 (23–183)	0.355
FeNO (ppb)	12 (6–19)	10 (5–20)	11 (5–18)	0.533
Blood eosinophils (10^9^/l)	0.16 (0.12–0.28)	0.18 (0.09–0.28)	0.19 (0.09–0.27)	0.870
Blood neutrophils (10^9^/l)	3.7 (2.8–4.5)	3.6 (2.9–4.9)	4.2 (3.2–5.0)	0.169
Pre-bd FEV_1_%	92 (87–98)	86 (75–97)**	75 (61–89)***^,##^	<0.001
Pre-bd FVC %	103 (91–110)	96 (87–104)*	90 (79–104)***	<0.001
Pre-bd FEV_1_/FVC	0.75 (0.71–0.80)	0.75 (0.67–0.79)	0.69 (0.57–0.76) ***^,##^	0.001
Post-bd FEV_1_%	96 (91–102)	89 (79–98)**	81 (64–94)***^,##^	<0.001
Post-bd FVC %	102 (93–110)	96 (85–106)	95 (83–105)**	0.007
Post-bd FEV_1_/FVC	0.77 (0.73–0.83)	0.76 (0.69–0.81)	0.71 (0.61–0.78)***^,##^	<0.001
Dl_CO_, %	96 (14)	96 (17)	87 (21)*^,#^	0.007
Dl_CO_/VA, %	94 (13)	98 (16)	93 (20)	0.126
AQ20 score	2 (0–4)	4 (2–6)***	8 (5–11)***^,###^	<0.001
ACT score	24 (22–25)	22 (20–24)***	18 (14–21)***^, ###^	<0.001
Baseline 15D score^†^	0.903 (0.089)	0.879 (0.075)	0.838 (0.109)**	0.001

Shown are mean (SD) or median (25–75 percentiles). Group comparisons performed by one-way ANOVA with Tukey’s post test (age, BMI, Dl_CO_, Dl_CO_/VA, Baseline 15D), Kruskal-Wallis test adjusted by Bonferroni correction for multiple tests (pack years, no of drugs in use to treat co-morbidities, no of add-on drugs, laboratory parameters, lung functions, AQ20 and ACT scores) or χ^2^–test with comparison of column proportions by z test and adjusting p-values by Bonferroni method (all categorical variables). *^,^** and *** indicates p < 0,05, p < 0,01 and p < 0,001 vs controlled group. ^♯,♯♯^ and ^♯♯♯^ indicates p < 0,05, p < 0,01 and p < 0,001 comparison to partially controlled group. ^&^Long-acting beta2-agonist, leukotriene receptor antagonist, theophylline and tiotropium (if not a COPD patient) included. FeNO = fraction of nitric oxide in exhaled air, BMI = Body Mass Index, bd = Bronchodilator, FVC = Forced vital capacity, LABA = Long-acting beta_2_-agonist, ICS = inhaled corticosteroid, Dl_CO_ = Diffusing capacity, Dl_CO_/VA = Diffusing capacity adjusted by the alveolar volume, AQ20 = Airways Questionnaire 20, ACT = Asthma Control Test. ^a^Part of the results have been previously published [7]. ^†^Information available on 171 patients.

### Generic HRQoL and asthma control

**Figure 2:**
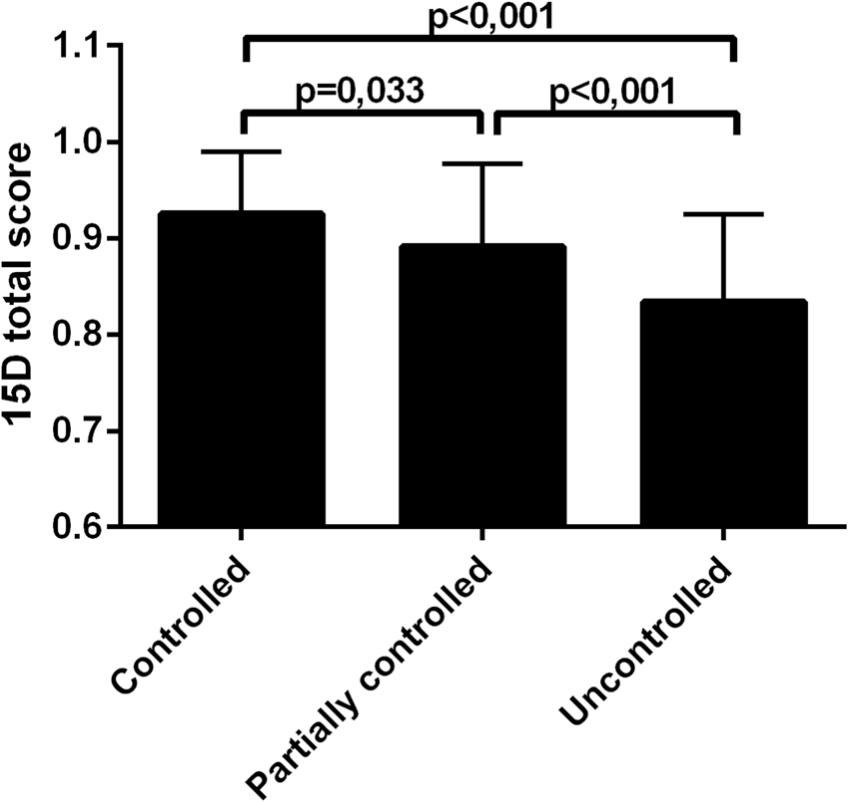
The mean 15D scores in asthma control groups. Shown are mean (SD) 15D scores. Group comparisons were performed by one-way ANOVA with Tukey’s post-test.

**Figure 3:**
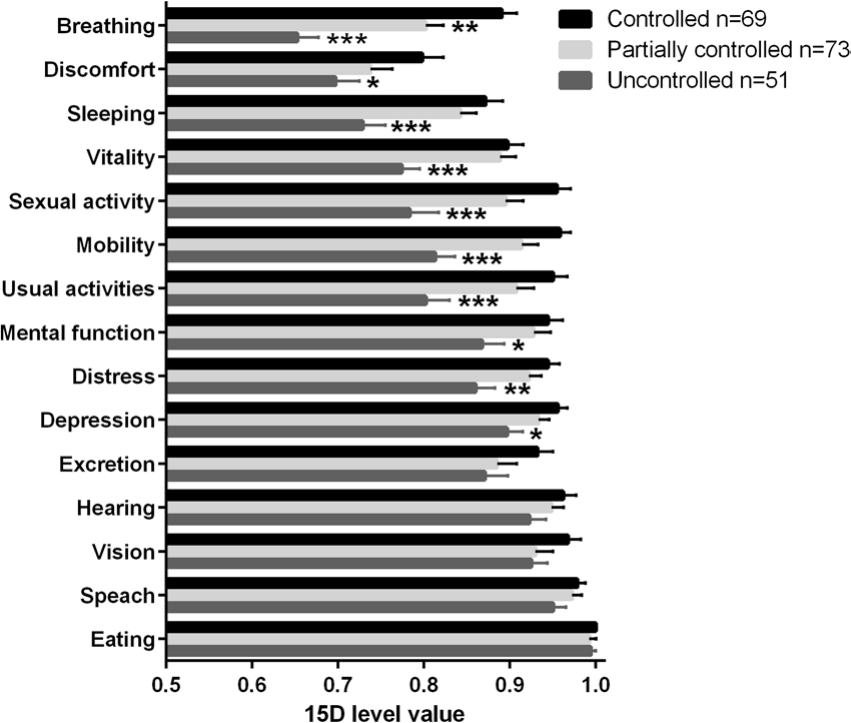
The mean 15D profiles of patients with uncontrolled and partially controlled asthma compared with patients with controlled asthma. Shown are mean scores and SEM of the 15D dimensions. *p < 0.05, **p < 0.01 and ***p < 0.001 as compared to patients with controlled asthma. Group comparisons (controlled, partially controlled, uncontrolled) were performed by one-way ANOVA with Tukey’s post-test.

Generic HRQoL, as measured by the 15D, was reduced in patients with uncontrolled and partially controlled asthma (defined according to GINA 2010^28^) compared to patients with controlled asthma (Fig. 2); the difference in the mean scores was −0.091 and −0.034, respectively. In addition, uncontrolled patients showed a lower level of generic HRQoL in comparison to partially controlled patients, the difference in the mean scores being 0.057. The mean differences between all groups were clinically important, i.e., exceeded the clinically important difference of ±0.015^27^. In more detail, there was a statistically significant difference between patients with controlled and uncontrolled asthma on 10 out of 15 dimensions; mobility, breathing, sleeping, usual activities, mental function, discomfort and symptoms, depression, distress, vitality and sexual activity were lower in patients with uncontrolled asthma (Fig. 3). Patients with partially controlled asthma differed from patients with controlled asthma only in the dimension of breathing (Fig. 3). Patients with uncontrolled asthma had a poorer 15D score at baseline (time of diagnosis) (Table 2).

For comparison, we used different definitions for asthma control, such as the recent GINA 2019 guideline^29^ where asthma control includes two components: symptom control and future risk of asthma exacerbations. When performing analysis according to the symptom control level of GINA 2019, the results remained almost identical (difference in the mean scores of uncontrolled and partially controlled asthma vs controlled asthma −0.092 and −0.038, respectively, 10/15 dimensions affected, Supplementary Fig. S1, S2A). Since it is not rational to compare current HRQoL in patients with and without future risk of exacerbations, we took into account the component of exacerbations by including data on recent history of exacerbations. When patients with at least two exacerbations during the previous two years were added into the uncontrolled group, the results remained mainly similar (difference in the mean scores of uncontrolled and partially controlled asthma vs controlled asthma −0.080 and −0.031, respectively, 8/15 dimensions affected, Supplementary Figs S1, S2B). Use of the ACT score to define asthma control resulted in an even larger difference in the 15D score between uncontrolled and controlled patients (difference in the mean scores −0.116 for uncontrolled and −0.034 for partially controlled vs. controlled, 11/15 dimensions affected, Supplementary Figs S1, S2C). To evaluate HRQoL in those patients with ≥2 severe exacerbations vs. those with 0–1 exacerbations during the previous two years, we carried out further analysis. The results revealed that patients with ≥2 exacerbations showed a lower 15D score than those with fewer exacerbations (difference in the mean score −0.060). Fewer dimensions were affected (5/15) compared to patients with uncontrolled asthma. Breathing, discomfort, mobility, usual activities and sexual activity (Supplementary Figs S1, S2D) were affected in patients with exacerbations but not mental function, depression, sleeping, distress or vitality. The two exacerbation groups did not show a difference in the baseline 15D score (0–1 exacerbations 0.880 (SD 0.088) vs ≥2 exacerbations 0.859 (SD 0.117), p = 0.294).

We included asthmatic smokers in this study and their number was highest in the group with uncontrolled asthma. Thus, a possibility exists that poorer HRQoL was seen in the uncontrolled group due to patients having ACO. To evaluate this possibility, we carried out further analyses by excluding patients who fulfilled the criteria of ACO (≥10 pack-years of smoking and postbronchodilator FEV_1_/FVC <0.7). After exclusion, the mean 15D score remained lower in the uncontrolled group as defined by GINA 2010 (controlled mean 0.924 (SD 0.067), partially controlled 0.899 (0.080), uncontrolled 0.834 (0.092), p < 0.001). The dimensions affected remained mainly similar, but the dimensions of depression and mental function lost significance after exclusion and were therefore mainly affected by ACO-patients. Very similar results were seen by using different definitions for asthma control after excluding patients with ACO (Supplementary Table S2).

In the whole cohort, the 15D score at the follow-up visit correlated with symptom measures (ACT rho = 0.499, p < 0.001, AQ20 rho = −0.576, p < 0.001) and baseline 15D score (rho = 0.567, p < 0.001) and showed a weak correlation with lung function measures (pre-BD FEV_1_ rho = 0.191, p<0.008 and post-BD FEV_1_ rho = 0.200, p < 0.005), BMI (rho = −0.245, p = 0.001) and age (rho = −0.277, p<0.001) but no correlation with traditional asthma biomarkers (FeNO, blood eosinophils, total IgE) or smoked pack-years.

### Factors associated with lower 15D score at follow-up

We evaluated factors associated with lower 15D score (poorer HRQoL) at follow-up using Tobit regression. A lower 15D score at the follow-up visit was associated with uncontrolled asthma, lower postbronchodilator FEV_1_, female sex, depression, treated dyspepsia and poorer 15D score at baseline. Partially controlled asthma, BMI, previous smoking and number of drugs in use (other indications than asthma/allergy) were not associated with poorer 15D score, even though the two latter had borderline significance (Table 3). Inclusion of exacerbations (≥2 during 2 previous years) into the model results in borderline significance (β-estimate −0.0260, 95% CI −0.0536 to 0.0017, p = 0.065) (Supplementary Table S3). Age of onset, smoked pack-years, current smoking, daily ICS treatment, blood eosinophils, neutrophils, FeNO, rhinitis, other comorbidities (diabetes, COPD, hypertension, coronary heart disease, any psychiatric disease, thyroid disorder, painful condition, hypercholesterolemia, rheumatic disease) or add-on drug in use for asthma did not explain the variance in the 15D score and were left out of the model.

**Table 3:** Predictors of 15D score at follow-up in Tobit regression.

Variable	Estimate	95% CI	*P*-value
Uncontrolled asthma	−0.0534	−0.0814 to −0.0254	<0.001
Partially controlled asthma	−0.0171	−0.0406 to 0.0064	0.153
Female gender	−0.0353	−0.0560 to −0.0146	0.001
Ex-smoker	−0.0210	−0.0424 to 0.0003	0.053
Number of drugs in use (indications other than asthma/allergy)	−0.0034	−0.0070 to 0.0002	0.067
Baseline 15D score	0.3260	0.2092 to 0.4427	<0.001
BMI	−0.0012	−0.0029 to 0.0005	0.180
post-BD FEV_1_ (% predicted)	0.0007	0.0001 to 0.0013	0.028
Treated dyspepsia	−0.0566	−0.0985 to −0.0146	0.009
Depression	−0.0402	−0.0789 to −0.0015	0.042

BMI = Body Mass Index, BD = Bronchodilator, FEV_1_ = Forced expiratory volume in 1 second, CI = confidence interval.

n = 166 due to missing values at baseline and follow-up 15D. Pseudo R^2^ = −0.398.

## Discussion

This study shows that the generic HRQoL measured by the 15D score was reduced in patients with uncontrolled asthma compared to patients with controlled disease. The difference was clinically important. There was a statistically significant difference between the controlled and uncontrolled groups on 10 out of 15 dimensions of the 15D, the affected dimensions being mobility, breathing, sleeping, usual activities, mental function, discomfort and symptoms, depression, distress, vitality and sexual activity. This result supports our hypothesis that uncontrolled asthma affects everyday life in several aspects.

Based on previous reports^13,14,17–19^ and comorbidities such as obesity, depression and anxiety associated with adult asthma^30^, it was not unexpected that the mean 15D scores on dimensions such as mobility, breathing, sleeping, distress, mental function and depression were lower in patients with uncontrolled asthma. In contrast, a striking effect of uncontrolled asthma was on the performance of usual activities. In this respect, as well as in terms of the mean 15D profile the results of patients with uncontrolled asthma resemble closely those of chronic obstructive pulmonary disease (COPD)^31^. Even after excluding patients fulfilling the criteria of ACO, patients with uncontrolled asthma showed poorer HRQoL with mainly similar affected dimensions. Interestingly, exclusion of ACO resulted in a loss of significance for mental function and depression, suggesting that uncontrolled patients with asthma-COPD overlap may have more problems related to HRQoL on these dimensions compared to patients with uncontrolled asthma only. Our study was underpowered for this comparison. Patients with ACO have been found to suffer from more comorbidities in general^32^ and have reported more frequent mental distress than patients with asthma alone^33^, but further studies are needed on the prevalence of depression and mental problems in asthma compared to ACO.

Surprisingly, there was a marked effect of uncontrolled asthma on vitality and sexual function. This is supported by a recent Spanish study^34^ reporting a poorer sexual QoL in patients with asthma. In their study, the level of asthma control (controlled vs. uncontrolled) was associated with sexual QoL with borderline significance. Our findings also suggest that sexual quality of life is poorer in patients with more frequent exacerbations during previous years, which has not been previously shown. Our study expands the current knowledge by showing a significantly reduced sexual QoL in patients with uncontrolled asthma and in patients with exacerbations.

Before 2006, the assessment and treatment of asthma was based on the disease severity. Patients with more severe asthma have been shown to have reduced HRQoL using both asthma-specific and generic instruments^10,35^. A radical shift was seen in 2006 when the revised GINA guidelines involved the classification and therapy of asthma according to the level of control (well controlled, partially controlled and uncontrolled). The therapy of the patient should be upgraded until control is achieved. This new paradigm has since been adopted to most local/national guidelines, and it is guiding asthma therapy practically all over the world. The old severity-based treatment algorithms have been largely abandoned. However, a major step in adopting a new disease classification system is to show that it has an impact on the quality of life of the patients. It has been previously shown that uncontrolled/not well-controlled asthma reduces asthma-specific HRQoL^12–16^, a finding that was seen also in the present study, as AQ20 scores indicated a lower asthma-specific HRQoL in patients with uncontrolled or partially controlled asthma. In contrast, less is known about the effect of asthma control level on generic HRQoL. Several population-based studies suggest that the generic HRQoL is lower in patients with uncontrolled asthma compared with controlled asthma^13,14,17–19,36–38^. However, in these studies, the diagnosis of asthma is usually derived from symptoms or self-reported from a physician’s diagnosis. Furthermore, no distinction has been made based on the time of onset (childhood vs. adulthood) or phenotypes of asthma. Variable ways of determining asthma control have been used, and several studies report only comparisons between uncontrolled and controlled asthma but do not include partially controlled asthma. We show here for the first time in a clinically confirmed (both by respiratory specialist and lung function) cohort of patients with adult-onset asthma that uncontrolled asthma defined according to GINA 2010^28^ and GINA 2019^29^ is associated with clinically and statistically significantly lower generic HRQoL, and this supports the rationale behind the use of such classification in the guidelines. In fact, a Swedish study recently reported that patients with well-controlled asthma did not differ from non-asthmatic controls when generic HRQoL was evaluated by the SF-36 Health Survey^19^. This further supports the idea that good asthma control should be the aim of the therapy.

The difference in generic HRQoL assessed with 15D between patients with controlled and uncontrolled asthma was>0.09 which exceeds the clinically important difference (±0.015)^27^ by a factor of 6, suggesting that uncontrolled asthma has a significant deleterious effect on the HRQoL of the patients. Using either definition of control, GINA 2010 (including assessment of symptoms and lung function) or GINA 2019 (based solely on symptoms), a similar difference was found. The GINA 2019 definition also includes components for the future risk of exacerbations, and when we included those with a recent history of exacerbations in the uncontrolled group, the results ended up as closely similar. Depending on what definition was used for uncontrolled asthma, a statistically significant difference was seen in 8–11 out of 15 dimensions assessed, suggesting an effect on most aspects of human life. Patients with at least two exacerbations during the previous years also showed a poorer mean 15D score compared to those with fewer exacerbations, exceeding the clinically important difference 4-fold. Regarding exacerbation groups, smaller differences and fewer affected dimensions might be expected because of the longer time frame compared to asthma control/questions on symptoms (2 years vs. 4 weeks). Accordingly, in the adjusted model including exacerbations as independent variables, uncontrolled asthma had more profound effects on generic HRQoL compared to having several exacerbations during the previous two years. In a previous study, frequent exacerbators (≥2 exacerbations per previous year) were shown to have decreased asthma-specific QoL as assessed by SGRQ^39^, supporting the results of our findings. However, SGRQ is restricted to components of symptoms, activities that are limited by breathlessness, social functioning and psychological disturbances, whereas 15D measures a wider array of aspects of life.

The difference between partially controlled and controlled asthma (>0.034) exceeded the clinically important difference by a factor of 2.3, although in the adjusted analysis, the association with HRQoL did not reach statistical significance. As the only dimension of the 15D significantly deteriorated in patients with partially controlled asthma was the breathing component, it seems possible that the main driver of lower generic HRQoL in patients with partially controlled asthma is related to lung function and respiratory symptoms. This is further supported by the finding that asthma-specific HRQoL (measured by AQ20) was significantly reduced in patients with partially controlled asthma.

Several population-based studies have suggested that uncontrolled/not well-controlled asthma is associated with lower generic HRQoL^13,14,17–19,36–38,40^. In two studies^14,18^, the effect of asthma control level (controlled/partially controlled/uncontrolled), as measured by using GINA-based evaluation, on generic HRQoL was evaluated. In the cohort with symptom-based asthma^14^ or the cohort with self-reported physician diagnosis of asthma^18^, both partially controlled and uncontrolled asthma (defined according to GINA) were associated with statistically lower generic HRQoL as evaluated with EQ-5D-3L^18^ or SF-36^14^. However, the differences as evaluated by the EQ-5D-3L^18^ or in most health domains of SF-36^14^ did not reach the level of accepted minimally important differences. The difference between their studies and the current study may be due to several factors. First, their cohorts were population-based cohorts with symptom-based definitions of asthma or patients self-reporting a physician’s diagnosis of asthma who were recruited by telephone, whereas the patients in our cohort had clinically confirmed asthma with verification of diagnosis both with lung function measurements and by specialist judgement^7,21^, and they were treated according to guidelines for 12 years^41^. This may mean that even though these cohorts look similar in many respects, patients in our cohort may represent more severe persistent asthma, and some patients with mild/intermittent asthma may have been excluded. This can also be considered a limitation of our study. However, our cohort represents almost 40% of the total cases of adult-diagnosed asthma in the geographical area^42^. Another difference is that patients in our cohort represent asthma diagnosed and starting at adult age and include several phenotypes (including smoking) with unfavourable long-term prognoses^4,32^, whereas previous studies also included patients with childhood-onset asthma. Additionally, both EQ-5D-3L and SF-36 cover fewer aspects of health than the 15D, and contrary to the 15D, they do not include a dimension measuring breathing problems. In addition, the EQ-5D-3L showed a marked ceiling effect (56% of the patients obtained a score of 1, suggesting full health)^18^. This casts doubt on the discriminatory power, credibility and thus the validity of the EQ-5D-3L in this patient group.

We found that a low 15D score was associated with two comorbidities, depression and treated dyspepsia, and showed borderline association with the number of drugs used to treat comorbidities. This is in line with previous studies reporting an association between depression or psychiatric disorders and lower QoL in patients with asthma as assessed by the 15D^20^, AQLQ^43^, SGRQ and SF-36^44^. To further support the association between depression and QoL, patients with aspirin-exacerbated respiratory disease (AERD) showed fewer depressive symptoms and better quality of life compared to other asthma patients, though no difference was seen in asthma control^45^. In previous studies, GERD was associated with poorer generic (SF-36)^46^ and asthma-specific (mini-AQLQ) QoL^47^ in both elderly asthmatics and adult patients from respiratory clinics, consistent with our findings. GERD was shown to have an effect on all domain scores of SF-36 in patients with asthma^46^. An important difference between our study and previous study was the definition of GERD, which was based on a symptom questionnaire in the previous study^46^ and medication in our study. Therefore, our results imply that even when treated, GERD still predicts poorer generic HRQoL in patients with asthma suggesting that GERD may still be undertreated in these patients. A connection between multimorbidity and HRQoL evaluated by the EQ-5D-3L has been previously reported^18^, and the association between the number of tablet medications and the HRQoL physical component as evaluated by SF-12^40^, also supporting our results.

Lower 15D score was significantly associated with female sex in our cohort of adult-onset asthma. Poorer generic and disease-specific HRQoL in asthmatic females compared to men were also shown before by using the SF-36, 15D and mini-AQLQ questionnaires^20,47,48^, and those results are not surprising, since asthmatic females have been shown to have poorer asthma control, more symptoms, poorer asthma-related quality of life, higher asthma-related healthcare utilization and a higher rate of depression, though with better lung function and a similar level of asthma severity, compared to men^15,48,49^. Generic HRQoL showed a high correlation with subjective disease-specific HRQoL (AQ20) and symptoms as measured by ACT but a much weaker correlation with objective measures of asthma such as lung function parameters, consistent with a previous study^50^. However, an association was found between post-BD FEV_1_ <80% and poorer 15D score in the adjusted model, suggesting that lung function has at least some significance for how patients actually feel or are able to function on a daily basis. Previously, it was reported that FEV_1_ <80% is associated with poorer score in the physical component summary of SF-36 but not mental component summary, suggesting that the association may depend on what dimensions are present or emphasized in the questionnaire^51^. Smoking was found to be associated with poorer 15D score in a previous study^15^ and has been previously associated with uncontrolled asthma^7,15^. In our study, current smoking was not associated with 15D, but ex-smoking remained of borderline significance. This may be explained by the “healthy smoker effect”, meaning that those who have quit smoking may be those who have suffered more respiratory symptoms and those who are able to continue smoking suffer less from smoking-related respiratory symptoms. An association between BMI and generic HRQoL was not shown in patients with asthma, consistent with a previous study using the 15D^20^. Our results showed a high correlation between diagnostic and follow-up 15D scores as well as a poorer 15D score at the time of diagnosis in patients with uncontrolled asthma. However, this finding does not explain the poorer HRQoL in patients with uncontrolled asthma, since the association remained even after including baseline 15D score in the same model. In a previous study with asthmatic adults, work-related overcommitment was reported as a predictor of asthma-related quality of life^52^. As approximately half of the patients in our cohort were in working life, work-related psychological stress would have been an interesting variable to add, but was not assessed in our study.

Our cohort represents patients with asthma diagnosed at adult age and to our knowledge, no other studies concentrating on a similar population of asthmatics and generic HRQoL exist, but we found a few studies on quality of life in early-onset vs. late-onset asthma. One study concentrating on older adults (>65 yrs) reported poorer asthma-specific quality of life (mini-AQLQ) associated with earlier onset asthma (<40 yrs) and poorer adherence in this group was speculated as one possible explanation^47^. Another study compared older patients with asthma onset>40 vs ≤40 yrs and found no difference in quality of life (mini-AQLQ), even though patients with early-onset asthma were more likely to have poor lung function or obstruction^53^. Our study found no significant role for age of onset in predicting generic HRQoL in a cohort with age of asthma onset varying between 15 and 77 yrs.

Given that both EQ-5D-3L and SF-36 scores^14,18^ have previously been associated with the level of asthma control, it would be tempting to use them as endpoints in clinical studies evaluating the effect of new therapies on asthma control and generic HRQoL. However, the differences between HRQoL levels in groups with different asthma control, even when comparing patients with controlled and uncontrolled asthma, most often has not reached clinically significant levels^14,18^. Furthermore, HUI-3, EQ-5D-3L and SF-6D have been reported to be able to differentiate between the highest and lowest levels of self-reported asthma control, but they could not discriminate between the moderate levels^36^. In the present study, 15D was able to discriminate well between controlled and uncontrolled asthma, and even the difference between controlled and partially controlled asthma was larger than the minimum clinically important difference^27^. This suggests that 15D may be the most useful tool for studies evaluating the effect of a given therapy on asthma control and generic HRQoL.

Taken together, our results show that in clinically confirmed patients with adult-onset asthma, the level of asthma control is associated with generic HRQoL evaluated by the 15D. Uncontrolled asthma was associated with lower generic HRQoL and with a HRQoL reduction on 10 out of 15 dimensions of the 15D: mobility, breathing, sleeping, usual activities, mental function, discomfort and symptoms, depression, distress, vitality and sexual activity. These results suggest that uncontrolled asthma significantly affects HRQoL and that the effects are not limited to respiratory organs.

## Supplementary information

Supplementary Data

## Data Availability

All data generated or analyzed during this study are included in this published article (and its Supplementary Information File). According to ethical permission and patient data-protection laws of Finland, single patient data cannot be made available.
