# Supplementary material for: Effect of asthma control on general health-related quality of life in patients diagnosed with adult-onset asthma
Source: Sci Rep. 2019 Nov 6;9:16107. doi: 10.1038/s41598-019-52361-9 (PMC6834611; doi:10.1038/s41598-019-52361-9)
Supplement: Supplementary file 1 — Supplementary Data [file 41598_2019_52361_MOESM1_ESM.pdf]

Supplementary Information

Effect of asthma control on general health-related quality of life in patients diagnosed with adult-onset asthma

Pinja Ilmarinen, PhD, Hind Juboori MD, Leena E. Tuomisto, MD, PhD, Onni Niemelä, MD, PhD, Harri Sintonen, PhD, Hannu Kankaanranta, MD, PhD

Table S1. The inclusion and exclusion criteria of SAAS study [e1]

Inclusion criteria	• a diagnosis of new-onset asthma made by a respiratory specialist<br>• diagnosis confirmed by at least one of the following objective lung function measurements[µ]:<br>o FEV_1 reversibility in spirometry of at least 15 % and 200 mL<br>o diurnal variability (≥ 20 %) or repeated reversibility (≥ 15 % / 60 L / min) in PEF-follow-up<br>o a significant decrease in FEV_1 (15 %) or PEF (20 %) in response to exercise or allergen<br>o a significant reversibility in FEV_1 (at least 15 % and 200 ml) or significant mean PEF in response to a trial with oral or inhaled glucocorticoids<br>• symptoms of asthma<br>• age ≥ 15 years
Exclusion criteria	• physical or mental inability to provide signed informed consent<br>• diagnosis of asthma below the age of 15 years<br>• of note:<br>o patients with comorbidities, either other lung disease or any other significant disease were not excluded<br>o patients were not excluded because of smoking, alcohol use or any other lifestyle factor<br>o respiratory symptoms or any other disease during childhood was not a reason to exclude patients, but a diagnosis of asthma at age <15 years was an exclusion criteria

[µ]The objective lung function criteria reflect those of national and international guidelines valid in 1999-2002 and may not exactly follow those valid at the moment [e2, e3].

Supplementary table S1 references:

e1. Kankaanranta H, Ilmarinen P, Kankaanranta T, Tuomisto LE. Seinäjoki adult asthma study (SAAS): a protocol for a 12-year real-life follow-up study of new-onset asthma diagnosed at adult age and treated in primary and specialised care. NPJ Prim Care Respir Med 25 (2015) 15042

e2. Haahtela T, Lehtimäki L, Ahonen E, Harju T, Jartti T, Kankaanranta H et al. Astman käypä hoito [Update on current guidelines: asthma]. Duodecim 129 (2013) 994–995.

e3. Global Initiative for Asthma: Global Strategy for Asthma Management and Prevention. Updated 2017. http://www.ginasthma.org/. Accessed: October 10th 2017.

Table S2. Generic health-related quality of life (15D score) in asthma control groups in patients after exclusion of asthma-COPD-overlap.

Definition of control	**Controlled**	**Partially controlled**	**Uncontrolled**	**P value**
GINA 2010				
**Subjects**	64	62	33	
**Total 15D score**[a]	0.924 (0.067)	0.899 (0.080)	0.834 (0.092)***,##	<0.001
GINA 2019				
**Subjects**	78	58	23	
**Total 15D score**[b]	0.923 (0.073)	0.882 (0.083)**	0.837 (0.085)***	<0.001
GINA 2019+exacerbations				
**Subjects**	74	47	38	
**Total 15D score**[c]	0.925 (0.072)	0.887 (0.082)*	0.850 (0.087)***	<0.001
ACT score				
**Subjects**	123	24	13	<0.001
**Total 15D score**[d]	0.911 (0.076)	0.880 (0.072)	0.782 (0.083)***,##	
Exacerbations	**0-1/previous 2yr**		**≥2/previous 2yr**	
**Subjects**	135		23	0.002
**Total 15D score**[e]	0.905 (0.079)		0.849 (0.091)	

Shown are mean (SD). Group comparisons were performed by independent samples t-test (exacerbations) or one-way ANOVA with Tukey's post-test (all other). ACT=asthma control test, 15D=15 dimensions, GINA=Global Initiative for Asthma. *, ** and *** indicates p<0,05, p<0,01 and p<0,001 vs controlled group. ## indicates p<0,01 in comparison to partially controlled group.

[a]Affected dimensions breathing, mobility, sleeping, discomfort, distress, vitality, sexual activity, usual activity
[b]Affected dimensions breathing, mobility, sleeping, discomfort, distress, vitality, sexual activity, usual activity
[c]Affected dimensions breathing, mobility, sleeping, discomfort, sexual activity, usual activity
[d]Affected dimensions breathing, mobility, sleeping, discomfort, mental, vitality, sexual activity, usual activity, hearing, speech
[e]Affected dimensions breathing, mobility, discomfort, sexual activity, usual activity

Table S3. Predictors of total 15D score at follow-up in Tobit regression, including exacerbations as independent variable.

**Variable**	Estimate	95% CI	*P*-value
Uncontrolled asthma	-.0385	-.0668 to -.0102	0.008
Partially controlled asthma	-.0121	-.0349 to .0107	0.296
Female gender	-.0315	-.0522 to -.0108	0.003
Ex-smoker	-.0193	-.0400 to .0014	0.067
Number of drugs in use (indications other than asthma/allergy)	-.0039	-.0075 to -.0002	0.036
Baseline 15D score	.3523	.2390 to .4657	<0.001
BMI	-.0011	-.0028 to .0005	0.185
Post-BD FEV_1 (% predicted)	.0007	.0001 to .0014	0.019
Treated dyspepsia	-.0395	-.0827 to .0037	0.073
Depression	-.0352	-.0729 to .0024	0.066
≥2 Exacerbations/2 yrs	-.0260	-.0536 to .0017	0.065

BMI = Body Mass Index, BD = Bronchodilator, FEV_1 = Forced expiratory volume in 1 second, CI=confidence interval. n=163 due to missing values at baseline and follow-up 15D and exacerbations. Pseudo R^2=-0.424

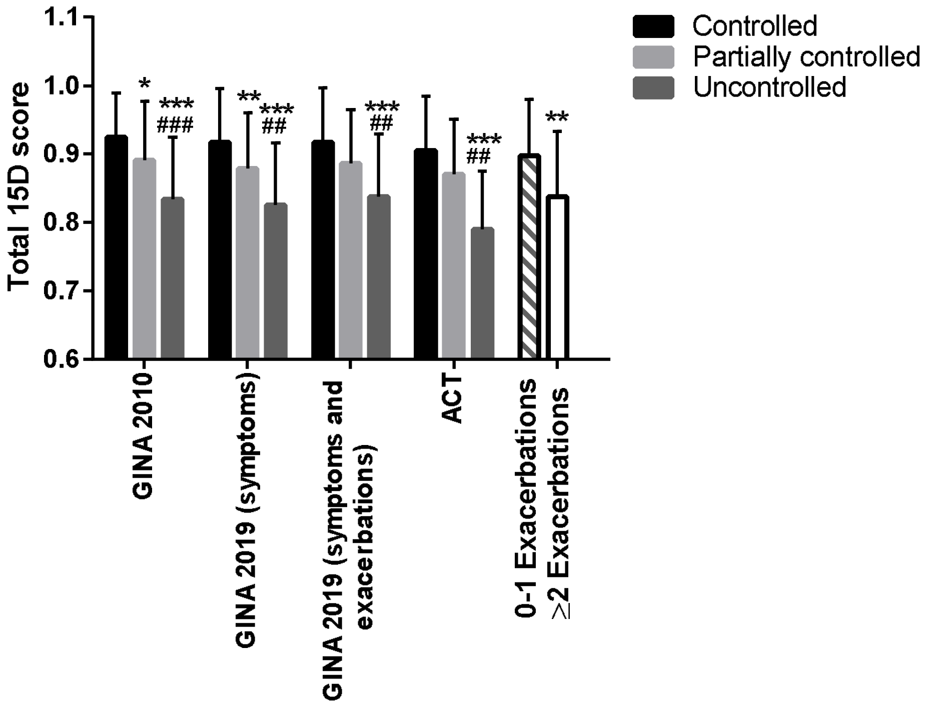

Figure S1. The mean 15D scores in asthma control/exacerbation groups. Asthma control was defined by different guidelines. Exacerbation groups are based on number of exacerbations during two previous years. Shown are mean (SD). Group comparisons were performed by one-way ANOVA with Tukey's post-test/independent samples t-test.

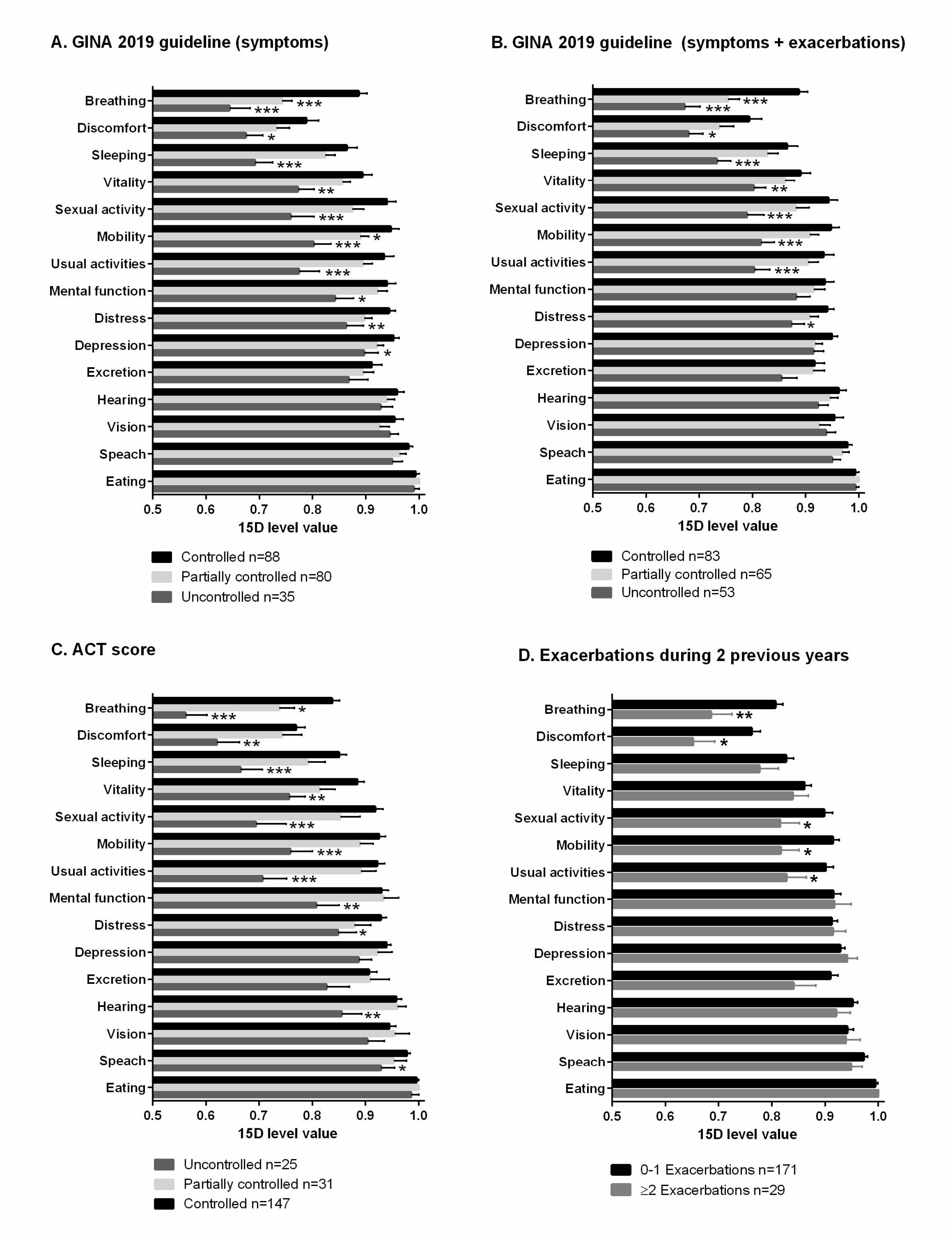

Figure S2. The mean 15D profiles of patients in asthma control groups by using different definition for asthma control (A-C) and in patients with recent history of severe exacerbations. Shown are mean scores and SEM of the 15D dimensions. *p<0.05, **p<0.01 and

***p<0.001 as compared to patients with controlled asthma (A-C) or patients with 0-1 exacerbations (D). Group comparisons performed by one-way ANOVA with Tukey's post-test (A-C) or by independent samples t-test (D).
